# 2-Aminoadipic acid (2-AAA) as a potential biomarker for insulin resistance in childhood obesity

**DOI:** 10.1038/s41598-019-49578-z

**Published:** 2019-09-20

**Authors:** Hyo Jung Lee, Han Byul Jang, Won-Ho Kim, Keon Jae Park, Kwang Youl Kim, Sang Ick Park, Hye- Ja Lee

**Affiliations:** 1 Center for Biomedical Sciences, National Institute of Health, Osong Health Technology Administration Complex, Chungcheongbuk-do, South Korea; 2 Department of Biochemistry, College of Medicine, Chungbuk National University, Chungcheongbuk-do, South Korea; 3 Department of Clinical Pharmacology, Inha University Hospital, Incheon, South Korea

**Keywords:** Cell signalling, Predictive markers

## Abstract

Insulin resistance is an important clinical feature of metabolic syndrome, which includes obesity and type 2 diabetes. Increased adipose energy storage in obesity promote insulin resistance and other metabolic adverse effects. To identify a new link between adipocyte and insulin resistance, we performed targeted metabolite profiling of differentiated adipocytes and studied the association between adipogenic metabolites and insulin resistance. We found a correlation between 2-aminoadipic acid (2-AAA) and adipogenic differentiation. Also, circulatory 2-AAA was positively associated with obesity-related factors (fat mass, fat percent, waist circumference, BMI, BMI *z*-score, triglycerides, insulin, and HOMA-IR) at baseline and after 2 years in the children cohort study. Of these factors, increased BMI *z*-score and HOMA-IR were the primary independent factors associated with higher 2-AAA levels, and the baseline 2-AAA level was an indicator of the BMI *z*-score after 2 years. To validate the relationship between 2-AAA and obesity-related factors, we analyzed changes in 2-AAA levels following obesity intervention programs in two independent studies. In both studies, changes in 2-AAA levels during the intervention period were positively correlated with changes in the BMI *z*-score and HOMA-IR after adjusting for confounders. Moreover, the 2-AAA levels were increased in cell and mouse models of obesity-related insulin resistance. Excess 2-AAA levels led to impaired insulin signaling in insulin-sensitive cells (liver, skeletal muscle and adipose cells) and caused abnormal gluconeogenesis. Our results demonstrate that 2-AAA is associated with adipogenesis and insulin resistance. In this regard, 2-AAA could be a potential biomarker of obesity and obesity-related metabolic disorders.

## Introduction

Obesity continues to increase in prevalence and has emerged as a serious public health problem worldwide. It is not simply a matter of appearance, as it also results in health risks because of associated physical and psychological problems^1,2^. In particular, one in three children is overweight or obese. Childhood obesity is likely to lead to adult obesity, which affects health during adulthood due to complications such as type 2 diabetes, hypertension, cardiovascular disease, and nonalcoholic fatty liver disease.

Obesity leads to insulin resistance, an important risk factor for the development of type 2 diabetes^3–5^. Obesity-induced insulin resistance is generally influenced by hypoxia, inflammation, and alteration of adipocytokine secretion. For example, excessive fat storage in adipose tissue results in hypertrophic adipocytes, causing inflammation in insulin-sensitive tissues, such as the liver and skeletal muscle. Moreover, inflammation reduces the abilities of insulin signaling pathway-related factors, such as insulin receptor substrate (IRS) and protein kinase B (AKT).

Recently, metabolite profiling was shown to be an important method for investigating the causes and possible treatments of various diseases. The levels of various circulating metabolites are altered in obesity, and weight loss reverses these alterations^6–10^. For example, increased branched-chain amino acids (BCAAs), aromatic amino acids, and anandamide are associated with insulin resistance, which is induced by obesity^6,7^. Asymmetric dimethylarginine (ADMA) and betaine have also been linked to risk of insulin resistance and type 2 diabetes, respectively^8–10^.

Among the metabolites evaluated, 2-aminoadipic acid (2-AAA) is produced by lysine degradation. Lysine residues are deaminated by metal-catalyzed oxidation to form allysine, which is oxidized to generate 2-AAA^11^. In previous studies, the levels of 2-AAA and other lysine pathway metabolites were significantly higher in diabetic mice. The level of allysine, a precursor of 2-AAA, was also increased in streptozotocin-induced diabetic rats^12,13^. In a metabolite profiling analysis of two cohorts with long follow-up periods, 2-AAA was associated with insulin resistance and markers predictive of an increased future risk of type 2 diabetes^14^.

As discussed above, altered levels of 2-AAA are important in the development of diabetes; however, whether they are also associated with obesity-related insulin resistance remains unclear. Therefore, we hypothesized that increased levels of 2-AAA are involved in adipogenesis and may be associated with insulin resistance. We evaluated whether the levels of 2-AAA were correlated with adipogenic differentiation at the cellular level and obesity-related insulin resistance in cross-sectional and follow-up cohort/intervention studies. Additionally, we sought to confirm the association between the levels of 2-AAA and insulin resistance using cell and mouse models. Next, we assessed whether accumulation of 2-AAA leads to insulin resistance, which could occur through impaired insulin signaling and abnormal gluconeogenesis.

## Results

### Association of 2-AAA levels with adipogenesis

Excess fat has been linked to many insulin resistance-related states, including obesity and type 2 diabetes^4,15^. Therefore, we investigated whether various metabolites were altered following the differentiation from preadipocytes to adipocytes. Cell lysates were extracted from subcutaneous preadipocytes and adipocytes (from a nondiabetic 39-year-old Caucasian woman with a body mass index (BMI) of 38 kg/m^2^), and the metabolite levels were measured. Following differentiation, metabolites with significantly decreased and increased levels were screened (Supplementary Table 1). Surprisingly, 2-AAA was detected in differentiated adipocytes but not in preadipocytes (Table 1). Thus, we found that alteration of 2-AAA levels was associated with adipogenesis.

**Table 1:** Quantities of 2-aminoadipic acid (2-AAA μM) in human preadipocytes and adipocytes.

	Preadipocytes	Adipocytes
Sample 1	Not Detected	1.27
Sample 2	Not Detected	1.40
Sample 3	Not Detected	1.18
Sample 4	Not Detected	1.57
Sample 5	Not Detected	1.51
Sample 6	Not Detected	0.86
Average	—	1.30 ± 0.26

### Association of 2-AAA levels with obesity and obesity-related factors in a cross-sectional study

**Figure 1:**
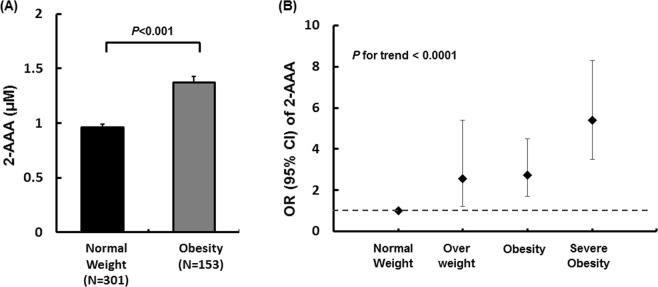
Association between plasma 2-aminoadipic acid (2-AAA) and obesity status in KoCAS-1. (**A**) Comparison of plasma 2-AAA levels between normal-weight and obese individuals and (**B**) odds ratios for 2-AAA levels according to obesity stage (overweight, body mass index [BMI] ≥ 23 and <25 kg/m^2^ or ≥85^th^ and <95^th^ percentile for age and sex [BMIp]; obese, BMI ≥ 25 and <30 kg/m^2^ or BMIp ≥ 95^th^ and <99^th^ percentile; severely obese, BMI ≥ 30 kg/m^2^ or BMIp ≥ 99^th^ percentile).

To evaluate the association of 2-AAA levels with obesity and obesity-related factors, we first examined 2-AAA levels according to obesity status (Supplementary Table 2, Fig. 1) in a cross-sectional study (KoCAS-1). The anthropometric measurements, lipid profiles, glycemic index, and 2-AAA levels were higher in the obese group than in the normal-weight group (all *p* ≤ 0.0356), whereas HDL-C levels were lower in the obese group (*p* < 0.0001). Obesity was significantly associated with increased 2-AAA levels in a stage-dependent manner (overweight: OR = 2.6, confidence interval [CI] = 1.2–5.5; obese: OR = 2.7, CI = 1.7–4.5; severely obese: OR = 5.4, CI = 3.4–8.4; *p* < 0.0001 for the trend).

The associations between plasma 2-AAA levels and obesity-related factors are shown in Table 2. Circulatory 2-AAA was positively associated with adiposity indices (fat mass, fat percent, waist circumference, BMI, and BMI *z*-score; all *p* ≤ 0.0336), lipid parameters (TG level; *p* < 0.0001), and glycemic parameters (glucose and insulin levels, HOMA-IR; all *p* ≤ 0.004) but was negatively correlated with the HDL-C level (*p* < 0.0001). According to the multiple linear stepwise regression results, the BMI *z*-score and HOMA-IR value were independent factors for high plasma 2-AAA levels (Table 2).

**Table 2:** Association of log-transformed baseline 2-AAA levels with obesity-related parameters in the Korean Children-Adolescents Study (KoCAS).

Variable	Simple regression			Stepwise regression^b^	
	Beta ± SE	*t*	*p*	Standardized beta	*p*
**KoCAS-1 (cross-sectional study)**					
**Adiposity parameters**					
Fat mass (kg)	0.011 ± 0.002	5.66	<0.0001		
Fat percentage (%)	0.005 ± 0.002	2.13	0.0336		
Waist circumference (cm)	0.014 ± 0.002	8.80	<0.0001		
BMI (kg/m^2^)	0.032 ± 0.004	7.80	<0.0001		
BMI *z*-score	0.133 ± 0.020	6.70	<0.0001	0.22	<0.0001
**Lipid parameters**					
Total cholesterol (mg/dL)	0.000 ± 0.000	0.69	0.4878		
HDL cholesterol (mg/dL)	−0.012 ± 0.002	−4.74	<0.0001		
Triglycerides (mg/dL)^a^	0.217 ± 0.048	4.54	<0.0001		
**Glycemic parameters**					
Fasting glucose (mg/mL)	0.013 ± 0.004	3.55	0.0004		
Insulin (μU/mL)^a^	0.276 ± 0040	6.92	<0.0001		
HOMA-IR^a^	0.272 ± 0.038	7.12	<0.0001	0.17	0.0002
**KoCAS-2 (2-year follow-up study, baseline 2-AAA vs follow-up obesity-related parameters)**					
**Adiposity parameters after 2-years**					
Fat mass (kg)	0.017 ± 0.003	5.21	<0.0001		
Fat percentage (%)	0.015 ± 0.003	5.12	<0.0001		
Waist circumference (cm)	0.014 ± 0.003	5.21	<0.0001		
BMI (kg/m^2^)	0.041 ± 0.007	5.76	<0.0001		
BMI *z*-score	0.179 ± 0.028	6.40	<0.0001	0.40	<0.0001
**Lipid parameters after 2-years**					
Total cholesterol (mg/dL)	0.000 ± 0.001	0.80	0.4261		
HDL cholesterol (mg/dL)	−0.003 ± 0.001	−2.57	0.0108		
Triglycerides (mg/dL)^a^	0.195 ± 0.058	3.36	0.0009		
**Glycemic parameters after 2-years**					
Fasting glucose (mg/mL)	−0.005 ± 0.004	−1.33	0.1839		
Insulin (μU/mL)^a^	0.171 ± 0.051	3.35	0.0010		
HOMA-IR^a^	0.143 ± 0.049	2.93	0.0038		

^a^Log-transformed before analysis.

^b^The variables included in the models were as follows: (1) KoCAS-1: age, sex, physical activity, pubertal stage, adiposity parameters (fat mass, fat percentage, waist circumference, body mass index [BMI], BMI *z*-score), lipid parameters (total cholesterol, high-density lipoprotein [HDL] cholesterol, triglycerides), and glycemic parameters (fasting glucose and insulin levels, homeostasis model assessment of insulin resistance [HOMA-IR]); (2) KoCAS-2: KoCAS-1 variables + baseline BMI.

### Replication of the results in the 2-year follow-up study

We next performed replication studies in independent subjects in the 2-year follow-up study (KoCAS-2; Supplementary Table 3). As in KoCAS-1, the baseline levels of 2-AAA were positively associated with baseline adiposity indices (fat mass, fat percent, waist circumference, BMI, and BMI *z*-score; all *p* ≤ 0.0001), lipid parameters (TG level; *p* = 0.0016), and glycemic parameters (insulin level and HOMA-IR; all *p* ≤ 0.0004) but were negatively correlated with the HDL-C level (*p* = 0.0105; Supplementary Table 4). The association between the baseline levels of 2-AAA and these obesity-related factors remained significant after 2 years (Table 2).

In the stepwise analyses, the baseline 2-AAA level was associated independently with the baseline BMI *z*-score, glucose level, and HOMA-IR (Supplementary Table 4). In particular, the baseline 2-AAA level was a major indicator of the BMI *z*-score after 2 years (Table 2).

### Intervention study

**Figure 2:**
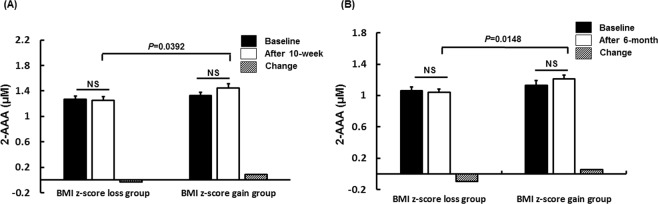
Plasma levels of 2-AAA according to the change in BMI *z*-score status in the (**A**) 10-week and (**B**) 6-month intervention studies. *p*_1_ values were obtained from paired *t*-tests. *p*_2_ values were calculated by analysis of covariance adjusted for the baseline 2-AAA level, age, sex, physical activity, and pubertal stage.

**Figure 3:**
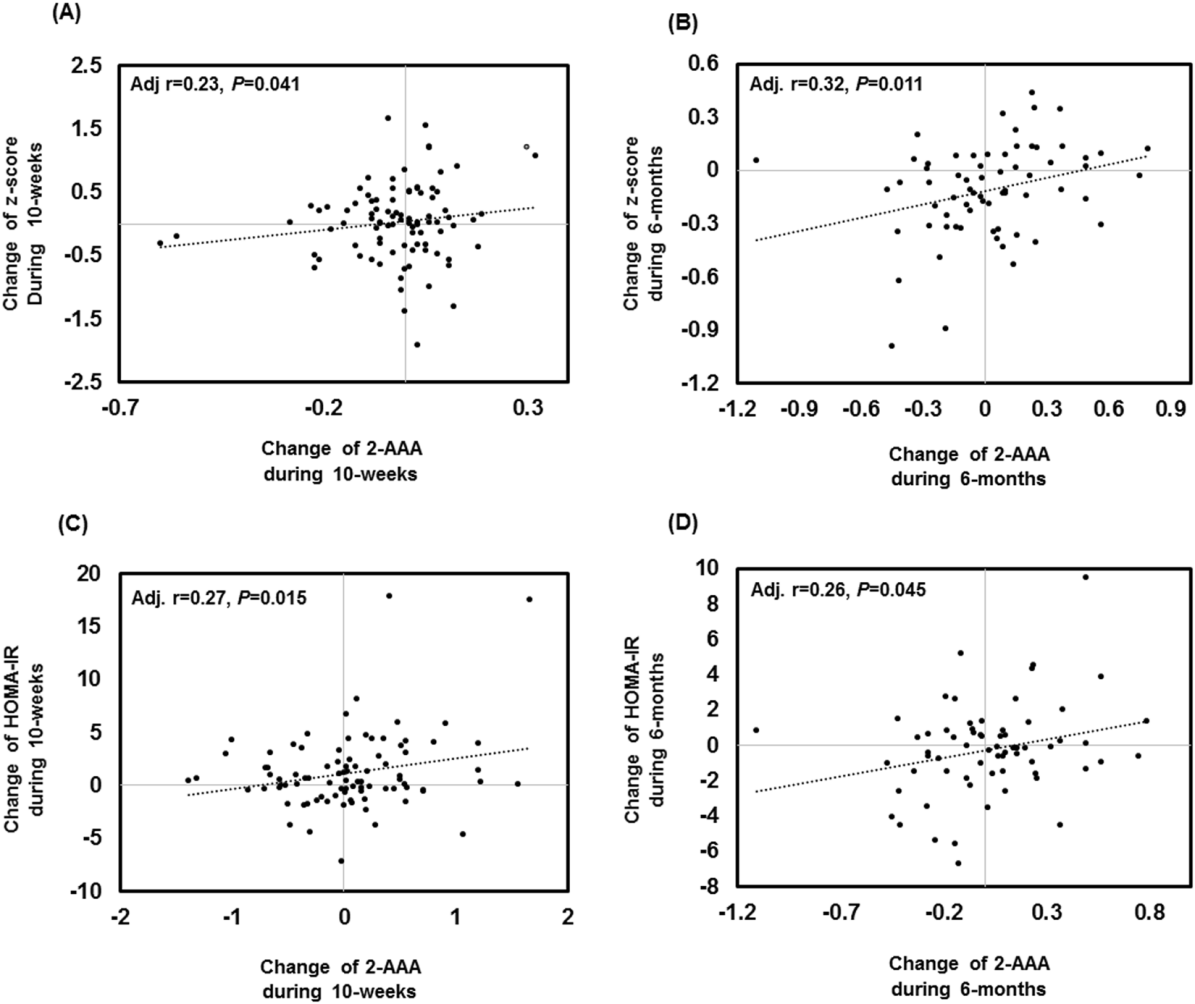
Correlations between changes in 2-AAA levels and obesity-related factors. Correlation between changes in 2-AAA levels and BMI *z*-scores in obese children after the (**A**) 10-week and (**C**) 6-month intervention programs. Correlations between changes in 2-AAA levels and homeostasis model assessment of insulin resistance (HOMA-IR) in the (**B**) 10-week and (**D**) 6-month intervention studies. Pearson’s partial correlation analyses were adjusted for age, sex, baseline BMI, physical activity, and pubertal stage.

To validate the relationship between the 2-AAA level and BMI *z*-score, we analyzed changes in 2-AAA levels following obesity intervention programs. In the 10-week intervention study, the BMI *z*-score loss group showed a decrease in the 2-AAA level following intervention completion compared with the BMI *z*-score gain group (*p* = 0.0392; Supplementary Table 5, Fig. 2A). Furthermore, after 10 weeks, decreased 2-AAA levels were correlated with a reduction in fat mass (*r* = 0.23, *p* = 0.044), BMI (*r* = 0.23, *p* = 0.037), BMI *z*-scores (*r* = 0.23, *p* = 0.041), TC levels (*r* = 0.32, *p* = 0.004), HDL-C levels (*r* = 0.26, *p* = 0.020), glucose levels (*r* = 0.29, *p* = 0.009), and HOMA-IR (*r* = 0.27, *p* = 0.015) after adjusting for confounders (age, sex, baseline BMI, physical activity, and pubertal stage; Supplementary Table 7, Fig. 3A,C).

The independent obesity intervention study also showed lower levels of 2-AAA in the BMI *z*-score loss group compared with the BMI *z*-score gain group following a 6-month intervention (*p* = 0.0148; Supplementary Table 6, Fig. 2B). The changes in 2-AAA levels during the 6-month period were positively correlated with changes in fat mass (*r* = 0.27, *p* = 0.038), BMI (*r* = 0.29, *p* = 0.025), BMI *z*-scores (*r* = 0.32, *p* = 0.011), HDL-C levels (*r* = 0.34, *p* = 0.008), and HOMA-IR (*r* = 0.26, *p* = 0.045) after adjusting for confounders (Supplementary Table 7, Fig. 3B,D).

### Alteration of 2-AAA levels by insulin resistance

**Figure 4:**
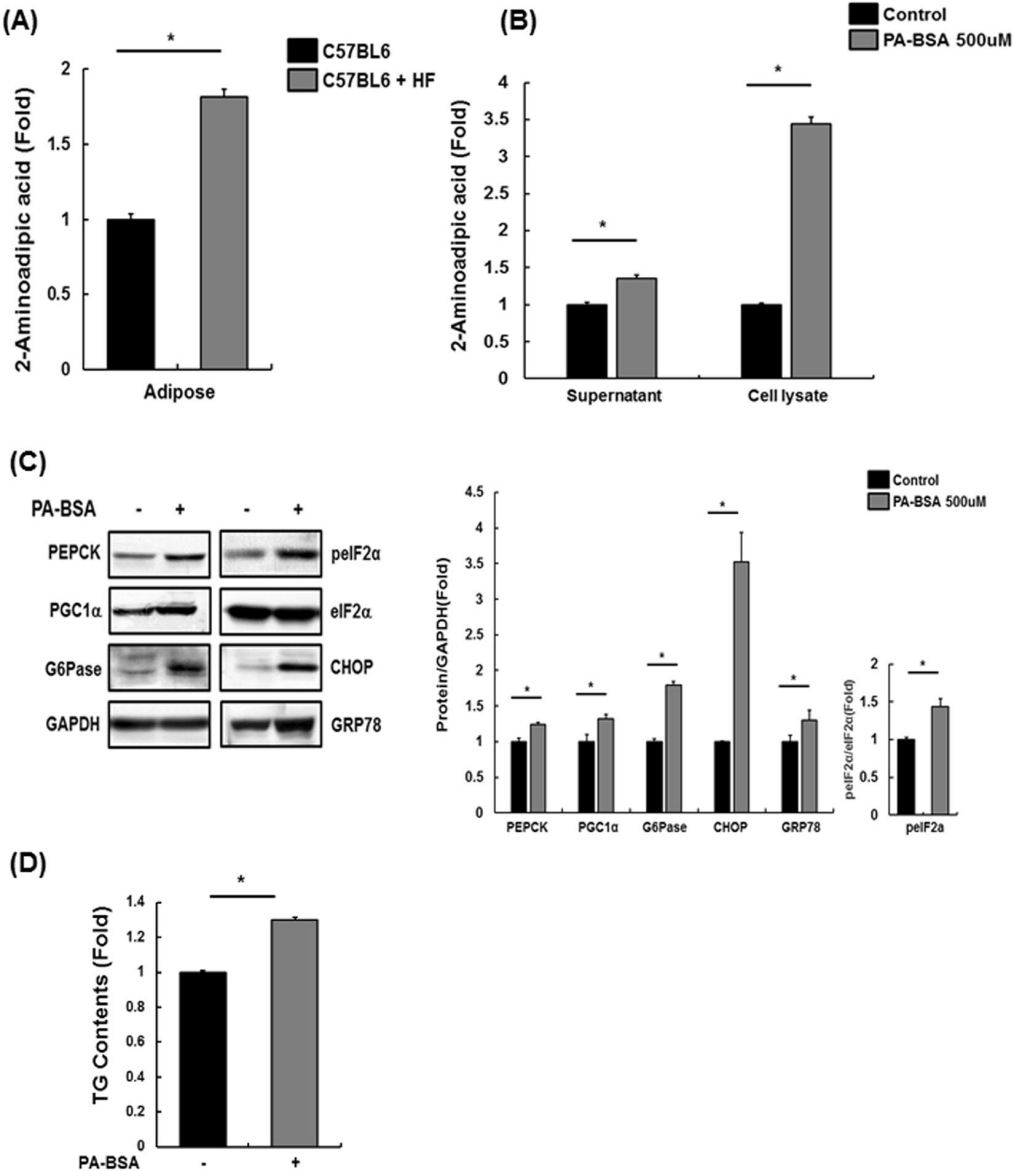
Excessive fatty acid-induced insulin resistance elevates 2-AAA levels. (**A**) Whole-cell lysates were extracted from adipose tissue of C57BL6 mice fed a high-fat diet and age-matched mice fed a standard diet, and 2-AAA levels were measured by liquid chromatography–tandem mass spectrometry. (**B**) 2-AAA levels were assessed in SK-Hep I cells treated with palmitate (PA; 500 μM) for 24 h. (**C**) Protein expression associated with the endoplasmic reticulum stress response and gluconeogenesis was analyzed in whole-cell lysates of SK-Hep I cells treated with PA (24 h, 500 μM) by western blotting. Middle-length blots and two exposures are presented in Supplementary Fig. 1. (**D**) Triglyceride (TG) levels measured in SK-Hep I cells treated with PA (500 μM) for 24 h. *Significant differences between groups at the *p* < 0.05 level.

Chronic consumption of a high-fat diet is closely related to obesity and insulin resistance^16,17^. Whole proteins were extracted from the adipose tissues of C57BL6 mice fed a high-fat diet and from age-matched mice fed a standard diet, and the levels of 2-AAA were assessed. As expected, the high-fat diet-fed mice exhibited higher body weight and glucose tolerance test (GTT) scores than the standard diet-fed mice (data not shown). Interestingly, 2-AAA levels were elevated in the adipose tissue of mice fed a high-fat diet compared with mice fed a standard diet (Fig. 4A). Palmitic acid (PA) mediates abnormal gluconeogenesis by inducing endoplasmic reticulum (ER) stress and insulin resistance^18,19^. We confirmed that ER stress markers (peIF2α, CHOP, and GRP78) and gluconeogenesis-related factors (PEPCK, PGC1α, and G6Pase) were increased in PA-treated SK-Hep I human liver cells (Fig. 4C, Supplementary Fig. 1). Additionally, excess PA resulted in elevated TG levels compared with the control (Fig. 4D). Remarkably, the levels of 2-AAA were higher in both lysates and supernatants from SK-Hep I cells treated with PA (Fig. 4B). These results suggest that increased levels of 2-AAA are associated with insulin resistance, which induces abnormal gluconeogenesis.

### Impaired insulin signaling by increased levels of 2-AAA

**Figure 5:**
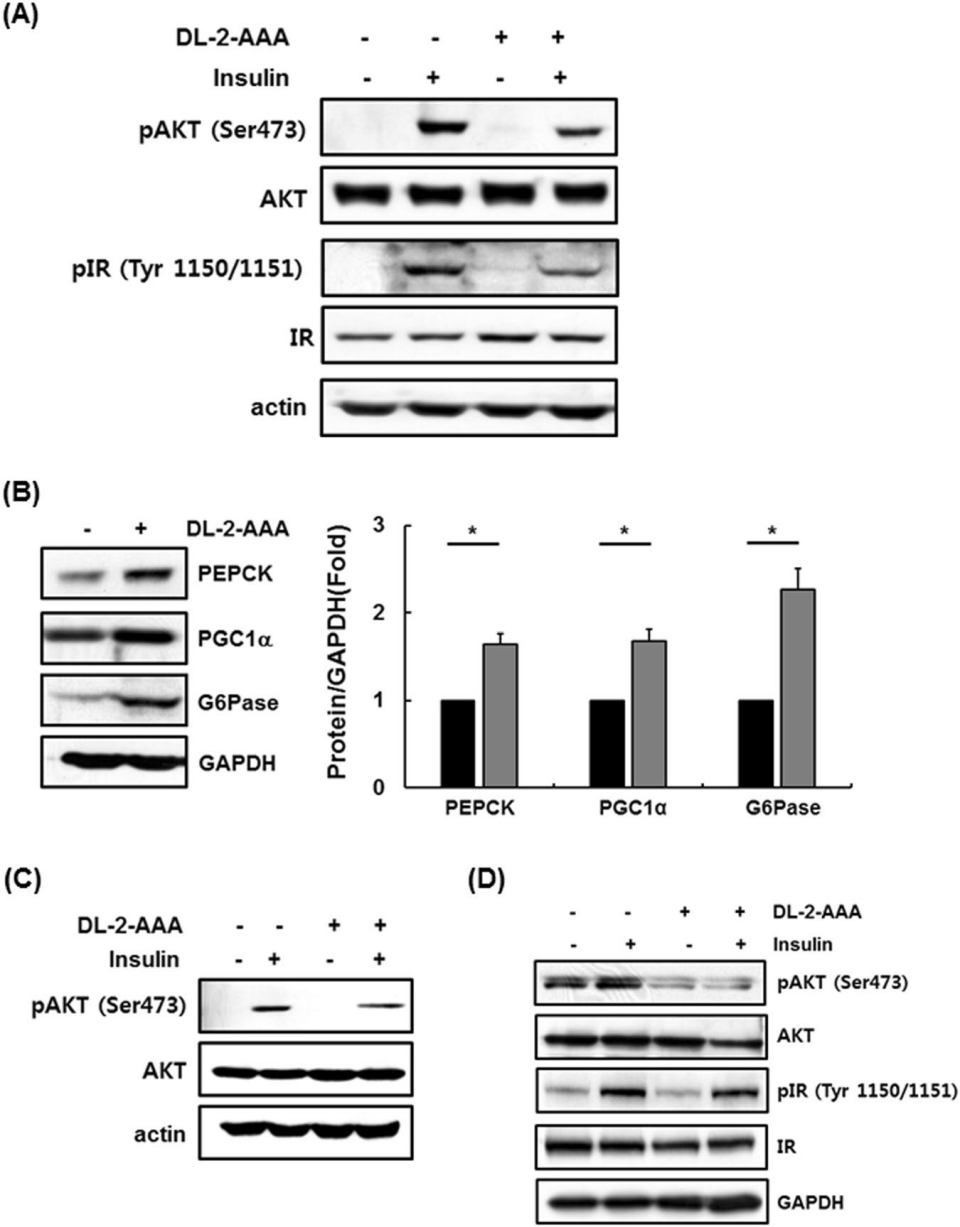
2-AAA accumulation induces insulin resistance. (**A,B**) SK-Hep I cells treated with DL-2-AAA (10 μM) for 24 h. The levels of proteins related to the insulin signaling pathway and gluconeogenesis were analyzed in whole-cell lysates by western blotting. (**C**) C2C12 myotubes treated with DL-2-AAA (10 μM) for 24 h. The levels of proteins associated with the insulin signaling pathway were analyzed in whole-cell lysates by western blotting. (**D**) Human subcutaneous adipocytes treated with DL-2-AAA (10 μM) for 24 h. The levels of proteins related to the insulin signaling pathway were analyzed in whole-cell lysates by western blotting. *Significant differences between groups at the *p* < 0.05 level. Middle-length blots and two exposures are presented in Supplementary Fig. 2.

In this study, increased 2-AAA levels were correlated with insulin resistance and obesity (Table 2, Figs 1–3). Additionally, we observed that 2-AAA levels were related to adipogenesis and increased in animal and cell models of induced insulin resistance (Table 1, Fig. 4). Therefore, excessive 2-AAA is a risk factor for insulin resistance. To explore the effect of 2-AAA on insulin signaling, SK-Hep I human liver cells, C2C12 mouse myotubes, and human subcutaneous adipocytes were treated with 2-AAA. Insulin signaling-related events, such as phosphorylation of AKT and phosphorylation of IR, were significantly reduced in 2-AAA-treated SK-Hep I cells (Fig. 5A, Supplementary Fig. 2A). At the same time, gluconeogenesis was significantly increased as a result of increased expression of PEPCK, G6Pase, and PGC1α (Fig. 5B, Supplementary Fig. 2B). As expected, AKT phosphorylation was decreased in 2-AAA-treated C2C12 mouse myotubes (Fig. 5C, Supplementary Fig. 2C). Treatment with 2-AAA caused a reduction in phosphorylation of AKT and a slight decrease in phosphorylation of IR in human subcutaneous adipocytes (Fig. 5D, Supplementary Fig. 2D). Overall, these results indicate that excess 2-AAA impairs insulin signaling. An increase in 2-AAA levels may contribute to the future development of diabetes due to abnormal gluconeogenesis.

## Discussion

Adipose tissue is an important organ involved in the regulation of insulin sensitivity and development of diabetes through its fat storage capacity and thermogenic regulation. However, when adipose tissue is no longer able to store fat, excess fat accumulates in ectopic lipid depots, such as the liver, intra-abdominal/visceral sites, and skeletal muscles. Increased ectopic lipid accumulation leads to dyslipidemia and insulin resistance^20–22^.

Insulin resistance is caused by impaired insulin signaling, and β cells promote abnormal production of insulin to maintain glucose homeostasis. When appropriate insulin secretion is not achieved in the pancreas, hyperglycemia and insulin resistance occur. Insulin resistance is an important clinical feature of metabolic syndrome, which includes obesity and type 2 diabetes^3–5,23^. Little is known about the metabolic mechanism involved in adipogenesis and insulin resistance. In this study, we differentiated human subcutaneous preadipocytes into adipocytes and performed metabolite profiling. Surprisingly, 2-AAA was detected in adipocytes but not in preadipocytes (Table 1). 2-AAA is produced primarily by oxidative stress and has been reported to be strongly linked to an increased risk of diabetes^11,14^. Thus, we generated a mouse model using C57BL6 mice in which insulin resistance was induced via the accumulation of excess fat; interestingly, 2-AAA levels were found to be elevated in the adipose tissue of these mice (Fig. 4A). We showed that excess palmitate plays an important role in the abnormal increase in gluconeogenesis by inducing the ER stress response and impairing insulin signaling^19^. As expected, the levels of 2-AAA were elevated in PA-treated SK-Hep I cells (Fig. 4B). Therefore, our results showed that 2-AAA is strongly correlated with insulin resistance.

The incidence of childhood obesity continues to increase globally and is a major public health challenge because it can promote many disorders in adulthood. It can also cause many acute health problems, including type 2 diabetes, early puberty, hypertension, musculoskeletal disorders, and psychological issues, leading to a considerable amount of suffering. Therefore, it is important to establish means for early prediction and preventive strategies for obesity because of the close correlation between childhood and adult obesity^1,2,24^. We identified a positive correlation of the plasma levels of 2-AAA with BMI and HOMA-IR in KoCAS. 2-AAA levels were significantly associated with obesity-related factors, including lipid parameters and HOMA-IR, at baseline and the 2-year follow-up. These results were validated in the obesity intervention study (Table 2, Fig. 3), in which changes in the 2-AAA levels during the intervention period were positively correlated with changes in the BMI z-score and HOMA-IR. Overall, we conclude that 2-AAA is associated with insulin resistance via elevated adipogenesis.

Previous studies have reported that the levels of 2-AAA were elevated in diabetic mice and human patients^12–14,25^. We demonstrated that 2-AAA levels were altered during adipogenesis and the insulin-resistant state (Table 1, Fig. 4). Since 2-AAA is likely involved in adipogenesis, it may also be associated with insulin resistance. This suggestion was confirmed by the observation that excess 2-AAA contributed to impaired insulin signaling. Insulin signaling was disturbed in 2-AAA-treated SK-Hep I human liver cells, C2C12 mouse myotubes, and human subcutaneous adipocytes (Fig. 5). Therefore, we suggest that excess 2-AAA is involved in impaired insulin signaling.

Recent studies have shown that BCAAs and aromatic amino acids are related to obesity-induced insulin resistance and that glutamate and BCAAs are associated with BMI^6,26,27^. Lipid metabolites reflecting dietary fatty acid intake have also been found to be correlated with obesity and insulin resistance, although some lipids exhibit differences according to race^28–30^. Previous research from our laboratory showed that ADMA is a potential biomarker for obesity-related insulin resistance^9^. Thus, the discovery of new biomarkers for disease can improve prediction and prevention in high-risk groups and establish new biological pathways associated with the onset of disease. Therefore, the identification of 2-AAA as a risk factor for childhood obesity and insulin resistance is noteworthy. Overall, our results demonstrate that 2-AAA is associated with adipogenesis and that altered levels of 2-AAA impair insulin signaling. Thus, 2-AAA could be a potential biomarker for insulin resistance-related obesity and may contribute to the early prevention of metabolic disorders, including obesity and type 2 diabetes.

## Materials and Methods

### Study population

The data were obtained from the Korean Children-Adolescents Study (KoCAS), conducted by the Korean National Institute of Health. The KoCAS-1 subjects were 449 adolescents aged 12–16 years from Seoul and Gyeonggi provinces for whom clinical biomarker data were collected between 2011 and 2012. The KoCAS-2 subjects were 200 independent individuals aged 9–11 years in 2008–2009, from whom health and metabolite data were obtained once more after 2 years. Obesity was defined as a body mass index (BMI) > 25 kg/m^2^ or being in the 95^th^ percentile for age and sex according to the 2007 Korean growth standard for children and adolescents.

For the short-term obesity intervention study, 88 morbidly obese adolescents aged 12–16 years with BMIs ≥ 99^th^ percentile (or ≥30 kg/m^2^) were recruited from Seoul and Gyeonggi provinces in 2012 as part of KoCAS. The intervention program included minimal exercise and nutrition education, in which the subjects participated three times over the course of 10 weeks. Data were also obtained from the Intervention for Childhood and Adolescent Obesity via Activity and Nutrition (ICAAN) study performed in 2016. The ICAAN study is an intensive multidisciplinary intervention program that includes exercise, nutrition education, and behavioral modification for obese children in Korea. In this study, we used data from 67 children aged 9–13 years with BMIs ≥ 90^th^ percentile who had completed the 6-month intervention program.

We obtained written informed consent prior to study participation from the parents of all subjects. All study protocols were approved by the institutional review board of Seoul-Paik Hospital, Inje University for the KoCAS (IIT-2009-071, IIT-2010-052, IIT-2011-058, and IIT-2012-092), Hallym University Sacred Heart Hospital for the ICAAN (2016-I135), and the Korea Center for Disease Control and Prevention (2017-02-06-P-A). The study procedures were carried out in accordance with approved guidelines.

### Metabolite profiling and 2-AAA quantitative analysis

Cell lysates extracted from human subcutaneous adipocytes (Caucasian woman, BMI of 38 kg/m^2^) and plasma from the KoCAS participants were analyzed using an AbsoluteIDQ p180 Kit (Biocrates Life Sciences AG, Innsbruck, Austria) according to the manufacturer’s instructions. The 2-AAA levels of cell lysates extracted from adipose tissue in mice, SK-Hep I cells, and plasma in ICAAN study participants were quantified by flow injection analysis–tandem mass spectrometry using an ABI 4000 Q-trap mass spectrometer (Applied Biosystems/MDS Sciex, Foster City, CA, USA) at the Inha University Hospital Clinical Trial Center.

### Clinical variables

Professionally trained personnel performed the anthropometric examinations using a standardized protocol. Height was measured with an automatic stadiometer (DS-102; Dong Sahn Jenix Co., Ltd., Seoul, South Korea), and body weight and composition were determined using a bioimpedance analyzer (BC-418; Tanita Corp., Tokyo, Japan) for KoCAS. In the ICAAN study, height was measured with a stadiometer (DS-103; Dong Sahn Jenix Co., Ltd.), and body weight and composition were measured using dual-energy X-ray absorptiometry (Lunar Prodigy Advance; GE Medical Systems Lunar, Madison, WI, USA). BMI was calculated as body weight in kilograms divided by squared height in meters (kg/m^2^) and converted into percentiles and *z*-scores based on the age- and sex-specific BMIs of the 2007 Korean national growth charts^31^. Waist circumference was measured at the midpoint between the lower border of the ribcage and iliac crest using a nonelastic tape measure.

Laboratory tests were performed only for individuals whose parents had agreed to the blood tests and provided written informed consent in advance. The concentrations of serum total cholesterol (TC), high-density lipoprotein-cholesterol (HDL-C), triglycerides (TG), and glucose were measured using enzymatic assays and an autoanalyzer (model 7600II; Hitachi, Tokyo, Japan). Fasting serum insulin was measured using a Roche E170 analyzer (Roche Diagnostics, Mannheim, Germany). The insulin resistance index was calculated using the homeostasis model assessment of insulin resistance (HOMA-IR)^32^.

Information on physical activity was collected using a self-reported questionnaire. Adolescents who met the national physical activity guidelines (moderate-intensity activity for ≥150 min/week on ≥5 days/week, or vigorous-intensity activity for ≥60 min/week on ≥3 days/week) were assigned to the active group. Pubertal development, determined using the method of Marshall and Tanner, was divided into three stages according to genitalia for boys and breasts for girls. We defined Tanner stage 1 as prepubertal, stages 2 and 3 as pubertal, and stages 4 and 5 as postpubertal development.

### Statistical analysis

All statistical tests were conducted using the SAS statistical software package (ver. 9.4; SAS Institute, Inc., Cary, NC, USA), and values are expressed as the means ± standard deviation (SD) for continuous variables and as percentages for categorical variables. Variables with nonnormal distributions (TG, insulin, HOMA-IR, 2-AAA) were log-transformed before the analysis. A paired *t*-test was used to comparatively assess altered metabolite levels between preadipocytes and adipocytes isolated from the same individual’s adipose tissues. The differences between the normal-weight and obese groups were tested using a general linear model adjusted for age, sex, physical activity, and pubertal stage. Logistic regression analysis was used to estimate odds ratios (ORs) for the presence of overweight, obesity, and severe obesity. Univariate linear regression and multivariate linear stepwise regression analyses were performed to assess associations between obesity-related clinical markers and plasma 2-AAA levels. The statistical significance of changes from baseline to after intervention program completion was assessed using a paired *t*-test. Analysis of covariance (ANCOVA) was used to compare differences among the BMI *z*-score groups following completion of the intervention programs after adjustment for baseline values, age, sex, physical activity, and pubertal stage. The association between changes in the 2-AAA level and changes in obesity-related factors during the obesity intervention period was assessed using partial correlation coefficients (*r*) controlling for age, sex, baseline BMI, physical activity, and pubertal stage. *P*-values < 0.05 were considered to indicate statistical significance.

### Chemicals and animals

Palmitate (PA), insulin, and DL-2-AAA were obtained from Sigma-Aldrich (St. Louis, MO, USA). To prepare PA-bovine serum albumin (BSA) solutions, PA was dissolved in ethanol and mixed with fatty acid-free BSA (2% w/v in water; Bovogen Biologicals, Keilor East, Australia) at 37 °C for 2 h with shaking. TG levels were determined using a TG quantification colorimetric/fluorometric kit (BioVision, Milpitas, CA, USA).

Male C57BL/6 mice were obtained from Japan Shizuoka Laboratory Animal Center (SLC). The mice were housed individually in plastic cages with bedding and allowed free access to a control chow diet and tap water. All mice were used for experiments at 5 weeks of age following an acclimation period of 1 week. The animals were divided into experimental groups matched for body weight and blood glucose levels. At 6 weeks of age, the mice were randomly fed a low-fat diet (10% energy from fat; D12450B, Research Diets, Inc., New Brunswick, NJ, USA) or a high-fat diet (60% energy from fat; D12492, Research Diets, Inc.) for 12 weeks. All animals were housed under standard light conditions (12-h light/dark cycle). Water was freely available throughout the experiment, and body weight was measured weekly.

The animal experiment protocols were approved by the Institutional Animal Care and Use Committee of Korea Centers for Disease Control and Prevention (KCDC-IACUC; KCDC-159-14-2A). The study procedures were carried out in accordance with approved guidelines.

### Cell culture

The SK-Hep I human liver cell line (CRL 1772; American Type Culture Collection, Manassas, VA, USA) was cultured in Dulbecco’s modified Eagle’s medium (DMEM) supplemented with 10% fetal bovine serum (FBS) and antibiotics. However, cells treated with PA-BSA solution (500 μM) or 2-AAA (10 μM) for 24 h were cultured in DMEM supplemented with 2% FBS and antibiotics. Cells treated with insulin were cultured in serum-free DMEM for 20 min.

Human subcutaneous preadipocytes were obtained from Zenbio (#SP-F-3; Research Triangle Park, NC, USA), cultured in preadipocyte medium (Zenbio), and differentiated using adipocyte differentiation medium (Zenbio) for 72 h.

### Western blotting

Following drug treatment, whole-cell lysates were prepared using lysis buffer (50 mM tris-buffered saline [Tris-Cl; pH 7.5], 20 mM NaCl, 5 mM ethylenediaminetetraacetic acid [EDTA], 1% Triton X-100, 0.1% sodium dodecyl sulfate, and 5% glycerol + protease inhibitor) and incubated on ice, followed by ultrasonication (Sonics & Materials, Inc., Newtown, CT, USA) for 10 s. Following centrifugation at 12,000 rpm for 20 min, the supernatants were separated by 7–12% sodium dodecyl sulfate–polyacrylamide gel electrophoresis and transferred to a polyvinylidene difluoride membrane. Following transfer, the membrane was blocked and probed with primary antibodies. The immunoblots were visualized using an enhanced chemiluminescence system (Thermo Fisher Scientific Inc., Rockford, IL, USA) and quantified using TINA 2.0 software (Raytest GmbH, Straubenhardt, Germany). Antibodies against phosphoenolpyruvate carboxykinase (PEPCK), peroxisome proliferator-activated receptor coactivator-1 alpha (PGC1α), glucose 6-phosphatase (G6Pase), and GRP78 were obtained from Santa Cruz Biotechnology (Dallas, TX, USA). Antibodies against eukaryotic initiation factor 2α (eIF2α), phospho (p)-eIF2α, CCAAT-enhancer-binding protein homologous protein (CHOP), insulin receptor (IR), p-IR, AKT, p-AKT, and glyceraldehyde 3-phosphate dehydrogenase (GAPDH) were obtained from Cell Signaling Technology (Beverly, MA, USA).

## Supplementary information

2-Aminoadipic acid (2-AAA) as a potential biomarker for insulin resistance in childhood obesity
