# Supplementary material for: 2-Aminoadipic acid (2-AAA) as a potential biomarker for insulin resistance in childhood obesity
Source: Sci Rep. 2019 Sep 20;9:13610. doi: 10.1038/s41598-019-49578-z (PMC6754510; doi:10.1038/s41598-019-49578-z)
Supplement: Supplementary file 1 — 2-Aminoadipic acid (2-AAA) as a potential biomarker for insulin resistance in childhood obesity [file 41598_2019_49578_MOESM1_ESM.docx]

2-Aminoadipic acid (2-AAA) as a potential biomarker for insulin resistance in childhood obesity

Hyo Jung Lee^1,2^, Han Byul Jang^1^, Won-Ho Kim^1^, Keon Jae Park^1^, Kwang Youl Kim^3^, Sang Ick Park^1^, Hye Ja Lee^1^

^1^Center for Biomedical Sciences, National Institute of Health, Osong Health Technology Administration Complex, Chungcheongbuk-do, South Korea

^2^Department of Biochemistry, College of Medicine, Chungbuk National University, Chungcheongbuk-do, South Korea

^3^Department of Clinical Pharmacology, Inha University Hospital, Incheon, South Korea

Hyo Jung Lee and Han Byul Jang contributed equally to this work.

Corresponding author. Division of Endocrine and Metabolic Diseases, Center for Biomedical Sciences, National Institute of Health, 187 Osongsaengmyung2-ro, Osong-eup, Heungdeok-gu, Cheongju-si, Chungcheongbuk-do, 28159, South Korea. Telephone: ++82-043-719-8692. Fax number: ++82-043-719-8602. E-mail: [hyejalee@yahoo.co.kr](mailto:hyejalee@yahoo.co.kr)

Supplementary Tables

Supplementary Table 1. List for differential analysis of metabolite profiles in human preadipocytes and adipocytes (p<0.05).^a^

| Direction | Classification | Metabolite |
| --- | --- | --- |
| N.D. → Detection | Biogenic Amines | 2-AAA |
| Increase | Amino-acids | Glutamin |
|  | Biogenic Amines | Putrescine, Spermidine |
|  | Acylcarnitines | C14:2-OH, C16, C16:1-OH, C18, C6:1, C9 |
|  | Glycerophospholipids LysoPC | lysoPC a C16:0, lysoPC a C16:1, lysoPC a C18:1, lysoPC a C28:1 |
|  | Glycerophospholipids PC | PC aa C28:1, PC aa C30:0, PC aa C30:2, PC aa C32:1, PC aa C32:2, PC aa C32:3, PC aa C34:1, PC aa C34:2, PC aa C34:3, PC aa C34:4, PC aa C36:2, PC aa C36:3, PC aa C38:3, PC aa C40:3, PC ae C30:1, PC ae C32:1, PC ae C32:2, PC ae C34:2, PC ae C34:3, PC ae C36:0, PC ae C36:3, PC ae C38:0, PC ae C38:6 |
|  | Sphingolipids Sphingomyelin | SM C18:0, SM C18:1, SM C24:1 |
|  | Hexose | H1 |
| Decrease | Amino-acids | Glutamate |
|  | Biogenic Amines | Spermine |
|  | Acylcarnitines | C12-DC |
|  | Glycerophospholipids LysoPC | lysoPC a C17:0, lysoPC a C18:0, lysoPC a C20:4 |
|  | Glycerophospholipids PC | PC aa C38:0, PC aa C38:4, PC aa C38:5, PC aa C38:6, PC aa C40:5, PC aa C40:6, PC aa C42:4, PC ae C30:0, PC ae C34:0, PC ae C34:1, PC ae C36:1, PC ae C36:4, PC ae C38:4, PC ae C38:5, PC ae C40:1, PC ae C40:4, PC ae C40:5, PC ae C40:6, PC ae C42:1, PC ae C42:2, PC ae C42:4, PC ae C44:3 |
|  | Sphingolipids Sphingomyelin | SM (OH) C14:1, SM C16:1, SM C26:1 |

Abbreviation: ND, not detected; PC, phosphatidylcholine

^a^To ensure data quality, each metabolite had to meet two criteria before analysis: a coefficient of variance of less than 15%, and 60% of the measured metabolite concentration in the experimental samples being above the limit of detection, which was set to three times the median value of the zero samples. A total of 186 plasma metabolites were measured, of which 119 met the above criteria.

Supplementary Table 2. The characteristics of the cross-sectional study (KoCAS-1) ^a^

|  | NW (N=301) | OB (N=148) | *P* ^b^ |
| --- | --- | --- | --- |
| Age (years) | 13.8 ± 0.4 | 13.8 ± 0.7 | 0.5681 |
| Sex (boy, %) | 43.9 | 52.0 | 0.1027 |
| Height (cm) | 161.4 ± 7.0 | 163.6 ± 8.2 | 0.0238 |
| Weight (kg) | 52.0 ± 6.7 | 84.6 ± 16.2 | <0.0001 |
| BMI (kg/m^2^) | 19.9 ± 1.8 | 31.4 ± 4.3 | <0.0001 |
| BMI z-score | -0.0 ± 0.6 | 2.4 ± 0.6 | <0.0001 |
| Waist circumference (cm) | 67.6 ± 5.9 | 95.9 ± 11.4 | <0.0001 |
| Fat percent (%) | 22.3 ± 8.0 | 41.1 ± 7.0 | <0.0001 |
| Fat mass (kg) | 11.7 ± 4.7 | 35.2 ± 11.4 | <0.0001 |
| Fasting glucose (mg/dL) | 93.2 ± 6.7 | 94.6 ± 8.1 | 0.0356 |
| Triglyceride (mg/dL) ^c^ | 73.8 ± 40.3 | 115.2 ± 61.9 | <0.0001 |
| Total cholesterol (mg/dL) | 158.5 ± 26.1 | 172.2 ± 30.6 | <0.0001 |
| HDL-cholesterol (mg/dL) | 54.9 ± 9.8 | 46.7 ± 8.5 | <0.0001 |
| Insulin (U/mL) ^c^ | 12.2 ± 7.8 | 25.1 ± 23.3 | <0.0001 |
| HOMA-IR ^c^ | 2.84 ± 1.89 | 5.98 ± 5.92 | <0.0001 |
| 2-AAA (μM) ^c^ | 0.96 ± 0.53 | 1.37 ± 0.69 | <0.0001 |
| Physical activity (active, %) | 66.1 | 57.4 | 0.0729 |
| Pubertal stage  (pre-PD/PD/post-PD, %) | 2.0/51.2/46.8 | 3.4/48.6/48.0 | 0.6287 |

Abbreviation: NW, normal weight; OB, obesity; BMI, body mass index; HOMA-IR, homeostasis model assessment of insulin resistance; 2-AAA, 2-Aminoadipic acid; PD, pubertal development.

^a^ Data were expressed as the mean±SD or %

^b^*P* value was calculated by generalized linear regression analysis with age, sex, physical activity, and pubertal stage for continuous parametric variables and Chi-square test for categorical variables.

^c^Variable was log transformed before analysis.

Supplementary Table 3. The characteristics of the 2-year follow-up study (KoCAS-2)

|  | Baseline (N=200) | Follow-up  (N=200) |
| --- | --- | --- |
| Age (years) | 10.4 ± 0.6 | 12.3 ± 0.7 |
| Sex (boy, %) | 53.0 |  |
| Height (cm) | 143.9 ± 7.1 | 156.0 ± 7.3 |
| Weight (kg) | 43.1 ± 9.8 | 53.5 ± 111.6 |
| BMI (kg/m^2^) | 20.7 ± 3.5 | 21.9 ± 3.8 |
| BMI z-score | 0.7 ± 1.0 | 0.7 ± 1.0 |
| Obesity (%) | 19.0 | 22.0 |
| Waist circumference (cm) | 68.9 ± 9.7 | 74.0 ± 10.1 |
| Fat percent (%) | 27.4 ± 9.0 | 27.0 ± 9.6 |
| Fat mass (kg) | 12.5 ± 6.6 | 15.2 ± 8.3 |
| Fasting glucose (mg/dL) | 84.9 ± 7.1 | 88.6 ± 7.8 |
| Triglyceride (mg/dL) | 89.0 ± 58.9 | 88.8 ± 46.6 |
| Total cholesterol (mg/dL) | 171.4 ± 27.6 | 167.8 ± 27.6 |
| HDL-cholesterol (mg/dL) | 54.7 ± 11.1 | 59.8 ± 21.8 |
| Insulin (U/mL) | 8.7 ± 7.0 | 13.7 ± 11.6 |
| HOMA-IR | 1.83 ± 1.59 | 5.98 ± 5.92 |
| 2-AAA (μM) | 0.84 ± 0.36 | 0.88 ± 0.41 |
| Physical activity (active, %) | 44.5 | 47.0 |
| Pubertal stage  (pre-PD/PD/post-PD, %) | 51.5/47.5/1.0 | 17.4/66.2/16.4 |

Abbreviation: BMI, body mass index; HOMA-IR, homeostasis model assessment of insulin resistance; 2-AAA, 2-Aminoadipic acid; PD, pubertal development.

Data were expressed as the mean±SD or %

Supplementary Table 4. Association of baseline 2-AAA with baseline obesity-related parameters in KoCAS-2.

| Variable | Simple regression | | |  | Stepwise regression ^b^ | |
| --- | --- | --- | --- | --- | --- | --- |
|  | Beta ± SE | t | *P* |  | Standardized beta | *P* |
| Adiposity parameters |  |  |  |  |  |  |
| Fat mass (kg) | 0.024 ± 0.004 | 5.79 | <0.0001 |  |  |  |
| Fat percent (%) | 0.019 ± 0.003 | 6.03 | <0.0001 |  |  |  |
| Waist circumference (cm) | 0.017 ± 0.003 | 5.86 | <0.0001 |  |  |  |
| BMI (kg/m^2^) | 0.048 ± 0.008 | 6.07 | <0.0001 |  |  |  |
| BMI z-score | 0.174 ± 0.029 | 5.97 | <0.0001 |  | 0.36 | <0.0001 |
| Lipid parameters |  |  |  |  |  |  |
| Total cholesterol (mg/dL) | 0.001 ± 0.001 | 1.51 | 0.1331 |  |  |  |
| HDL-cholesterol (mg/dL) | -0.007 ± 0.002 | -2.58 | 0.0105 |  |  |  |
| Triglyceride (mg/dL) ^a^ | 0.161 ± 0.050 | 3.21 | 0.0016 |  |  |  |
| Glycemic parameters |  |  |  |  |  |  |
| Fasting glucose (mg/mL) | -0.011 ± 0.004 | -2.53 | 0.0123 |  | -0.28 | <0.0001 |
| Insulin (μU /mL) ^a^ | 0.188 ± 0047 | 4.03 | <0.0001 |  |  |  |
| HOMA-IR ^a^ | 0.164 ± 0.046 | 3.60 | 0.0004 |  | 0.15 | 0.0478 |

^a^Variable was log transformed before analysis.

^b^The variables included in the models were: age, sex, physical activity, pubertal stage, adiposity parameters (fat mass, fat percent, waist circumference, , body mass index [BMI], BMI z-score), lipid parameters (Total cholesterol, high-density lipoprotein [HDL] cholesterol, triglyceride), and glycemic parameters (fasting glucose, insulin, homeostasis model assessment of insulin resistance [HOMA-IR])

Supplementary Table 5. The characteristics of 10-weeks intervention study. ^a^

|  | BMI z-score loss group (N=43) | | |  | BMI z-score gain group (N=45) | | | *P* ^c^ |
| --- | --- | --- | --- | --- | --- | --- | --- | --- |
|  | Pre | Post | *Difference* ^b^ |  | Pre | Post | *Difference* ^b^ |  |
| Age (years) | 14.0 ± 0.9 |  |  |  | 14.0 ± 0.7 |  |  | 0.7076 |
| Sex (boy, %) | 55.8 |  |  |  | 46.7 |  |  | 0.3908 |
| Height (cm) | 164.5 ± 7.4 | 164.5 ± 7.5 | 0.1 |  | 165.1 ± 7.4 | 165.1 ± 7.4 | 0.1 | 0.9536 |
| Weight (kg) | 92.1 ± 12.1 | 90.0 ± 12.7 | -2.1^***^ |  | 93.8 ± 14.6 | 95.9 ± 16.2 | 2.0^***^ | <0.0001 |
| BMI (kg/m^2^) | 34.0 ± 2.9 | 33.1 ± 3.1 | -0.8^***^ |  | 34.2 ± 2.9 | 34.9 ± 3.4 | 0.7^***^ | <0.0001 |
| BMI z-score | 2.8 ± 0.4 | 2.7 ± 0.4 | -0.1^***^ |  | 2.8 ± 0.3 | 2.9 ± 0.3 | 0.1^***^ | <0.0001 |
| Waist circumference (cm) | 103.3 ± 8.0 | 99.7 ± 8.4 | -3.7^***^ |  | 104.2 ± 10.3 | 102.9 ± 11.6 | -1.4 | 0.0665 |
| Fat percent (%) | 45.2 ± 6.1 | 44.3 ± 6.0 | -0.8 |  | 45.1 ± 4.2 | 46.8 ± 5.6 | 1.3^***^ | 0.0027 |
| Fat mass (kg) | 41.6 ± 8.3 | 40.1 ± 9.1 | -1.5^*^ |  | 42.0 ± 8.4 | 45.2 ± 12.7 | 1.9^***^ | <0.0001 |
| Triglyceride (mg/dL) ^d^ | 130.9 ± 65.0 | 110.3 ± 51.1 | -20.7^**^ |  | 135.6 ± 77.3 | 126.4 ± 61.4 | -9.2 | 0.1115 |
| Total cholesterol (mg/dL) | 177.3 ± 28.1 | 172.4 ± 31.8 | -4.9 |  | 180.7 ± 24.7 | 179.6 ± 27.6 | -1.1 | 0.2692 |
| HDL-cholesterol (mg/dL) | 44.7 ± 7.3 | 46.2 ± 9.7 | 1.5 |  | 44.2 ± 7.0 | 46.3 ± 7.1 | 2.1^*^ | 0.8034 |
| Fasting glucose (mg/dL) | 96.6 ± 24.1 | 99.9 ± 24.7 | 3.3 |  | 94.1 ± 11.6 | 99.1 ± 21.9 | 5.0^*^ | 0.6087 |
| Insulin (U/mL) ^d^ | 24.1 ± 8.2 | 26.7 ± 11.3 | 2.6 |  | 28.6 ± 14.3 | 32.2 ± 14.5 | 3.6^*^ | 0.2747 |
| HOMA-IR ^d^ | 5.7 ± 2.2 | 6.7 ± 3.3 | 0.1 |  | 6.8 ± 3.9 | 8.2 ± 5.8 | 0.2^*^ | 0.3479 |
| 2-AAA (μM) | 1.27 ± 0.41 | 1.25 ± 0.44 | 0.0 |  | 1.33 ± 0.56 | 1.45 ± 0.42 | 0.1 | 0.0392 |
| Physical activity (active, %) | 49.4 |  |  |  | 54.6 |  |  | 0.4536 |
| Pubertal stage  (pre-PD/PD/post-PD, %) | 4.7/37.2/58.1 |  |  |  | 8.9/57.8/33.3 |  |  | 0.0638 |

Abbreviation: BMI, body mass index; HOMA-IR, homeostasis model assessment of insulin resistance; 2-AAA, 2-Aminoadipic acid; PD, pubertal development.

^a^Data were expressed as the mean±SD or %.

^b^Difference was calculated by paired *t*-test: ^*^ *p* < 0.05, ^**^ *p* < 0.01, ^***^ *p* < 0.001

^c^*P* value was calculated by analysis of covariance(ANCOVA) after adjustment for baseline values, age, sex, physical activity, and pubertal stage for continuous parametric variables and Chi-square test for categorical variables.

^d^Variable was log transformed before analysis.

Supplementary Table 6. The characteristics of 6-month intervention study (ICAAN). ^a^

|  | BMI-z score loss group (N=42) | | |  | BMI-z score gain group (N=25) | | | *P* ^c^ |
| --- | --- | --- | --- | --- | --- | --- | --- | --- |
|  | Pre | Post | *Difference* ^b^ |  | Pre | Post | *Difference* ^b^ |  |
| Age (years) | 11.0 ± 1.0 |  |  |  | 11.4 ± 0.8 |  |  | 0.0778 |
| Sex (boy, %) | 28.6 |  |  |  | 44.0 |  |  | 0.1983 |
| Height (cm) | 152.8 ± 9.6 | 156.3 ± 9.5 | 3.5^***^ |  | 153.8 ± 7.2 | 157.2 ± 7.3 | 3.4^***^ | 0.5120 |
| Weight (kg) | 68.2 ± 15.1 | 68.8 ± 14.1 | 0.6 |  | 67.2 ± 11.3 | 73.3 ± 13.1 | 6.1^***^ | <0.0001 |
| BMI (kg/m^2^) | 28.9 ± 3.9 | 28.0 ± 3.8 | -0.9^***^ |  | 28.3 ± 2.9 | 29.5 ± 3.5 | 1.2^***^ | <0.0001 |
| BMI z-score | 2.4 ± 0.5 | 2.1 ± 0.6 | -0.3^***^ |  | 2.2 ± 0.5 | 2.4 ± 0.5 | 0.1^***^ | <0.0001 |
| Waist circumference (cm) | 90.4 ± 9.7 | 89.2 ± 9.3 | -1.2 |  | 88.1 ± 8.2 | 90.6 ± 9.3 | 2.5^**^ | 0.0276 |
| Fat percent (%) | 43.0 ± 4.4 | 42.6 ± 4.3 | -0.4^***^ |  | 41.6 ± 3.7 | 41.3 ± 3.7 | -0.3^***^ | 0.1347 |
| Fat mass (kg) | 29.3 ± 8.0 | 28.4 ± 7.5 | -0.9 |  | 27.7 ± 5.1 | 30.7 ± 6.3 | 3.0^***^ | <0.0001 |
| Triglyceride (mg/dL) ^d^ | 109.1 ± 40.8 | 106.1 ± 43.3 | -3.0 |  | 100.2 ± 46.2 | 124.0 ± 55.6 | 23.8^*^ | 0.1039 |
| Total cholesterol (mg/dL) | 177.2 ± 29.9 | 170.6 ± 26.0 | -0.3 |  | 164.0 ± 18.1 | 167.0 ± 24.1 | 5.5 | 0.3368 |
| HDL-cholesterol (mg/dL) | 49.7 ± 10.8 | 49.3 ± 9.2 | -0.4 |  | 51.9 ± 8.7 | 49.0 ± 9.5 | -2.9 | 0.1924 |
| Fasting glucose (mg/dL) | 90.1 ± 8.2 | 90.8 ± 6.8 | 0.6 |  | 86.6 ± 6.4 | 89.6 ± 7.5 | 3.0 | 0.8793 |
| Insulin (U/mL) ^d^ | 22.2 ± 10.9 | 18.0 ± 6.5 | -4.2^**^ |  | 20.5 ± 10.1 | 23.7 ± 12.3 | 3.2 | 0.0089 |
| HOMA-IR ^d^ | 5.0 ± 2.4 | 4.1 ± 1.6 | -0.9^*^ |  | 4.4 ± 2.5 | 5.3 ± 2.9 | 0.9 | 0.0163 |
| 2-AAA (μM) | 1.06 ± 0.25 | 1.04 ± 0.29 | -0.0 |  | 1.14 ± 0.34 | 1.24 ± 0.28 | 0.1 | 0.0148 |
| Physical activity (active, %) | 31.0 |  |  |  | 36.0 |  |  | 0.6705 |
| Pubertal stage  (pre-PD/PD/post-PD, %) | 41.5/58.5/0.0 |  |  |  | 28.0/72.0/0.0 |  |  | 0.2700 |

Abbreviation: BMI, body mass index; HOMA-IR, homeostasis model assessment of insulin resistance; 2-AAA, 2-Aminoadipic acid; PD, pubertal development.

^a^Data are expressed as the mean±SD or %.

^b^Difference was calculated by paired *t*-test: ^*^ *p* < 0.05, ^**^ *p* < 0.01, ^***^ *p* < 0.001

^c^*P* value was calculated by analysis of covariance(ANCOVA) after adjustment for baseline values, age, sex, physical activity, and pubertal stage for continuous parametric variables and Chi-square test for categorical variables.

^d^Variable was log transformed before analysis.

Supplementary Table 7. Correlation of changes of 2-AAA and obesity-related factors after obesity intervention program.

|  | 10-weeks intervention | | 6-months intervention | |
| --- | --- | --- | --- | --- |
|  | *r* | *p* | *r* | *p* |
| Adiposity |  |  |  |  |
| *Δ* Fat percent (%) | 0.19 | 0.091 | 0.25 | 0.054 |
| *Δ* Fat mass (kg) | 0.25^*^ | 0.025 | 0.27^*^ | 0.034 |
| *Δ* Waist circumference (cm) | 0.03 | 0.808 | 0.25 | 0.049 |
| *Δ* BMI (kg/m^2^) | 0.26^*^ | 0.020 | 0.29^*^ | 0.020 |
| *Δ* BMI z-score | 0.26^*^ | 0.017 | 0.33^*^ | 0.010 |
| Lipid parameters |  |  |  |  |
| *Δ* Total cholesterol (mg/dL) | 0.34^**^ | 0.002 | -0.38 | 0.775 |
| *Δ* HDL-cholesterol (mg/dL) | 0.29^*^ | 0.009 | 0.33^**^ | 0.008 |
| *Δ* Triglyceride (mg/dL) | -0.19 | 0.091 | -0.09 | 0.443 |
| Glycemic parameters |  |  |  |  |
| *Δ* Fasting glucose (mg/mL) | 0.30^**^ | 0.006 | 0.07 | 0.599 |
| *Δ* Insulin (μU/mL) | 0.27 | 0.016 | 0.26 | 0.045 |
| *Δ* HOMA-IR | 0.33^*^ | 0.003 | 0.28^*^ | 0.036 |

*Δ,* Change in value during the intervention period.

*P* value was assessed using Pearson’s partial correlation with adjustment for age, sex physical activity and pubertal stage. If theses correlations remained statistically significant after further adjustment of baseline BMI, they are marked with an asterisk (^*^ *P* < 0.05, ^**^ *P* < 0.01, ^***^ *P* < 0.001). The partial correlation coefficients (*r*) and p-value, additionally adjusted for baseline BMI, were stated in the results of the text.

Supplementary Figure 1. Middle-length blots and two exposures of Figure 4C

**
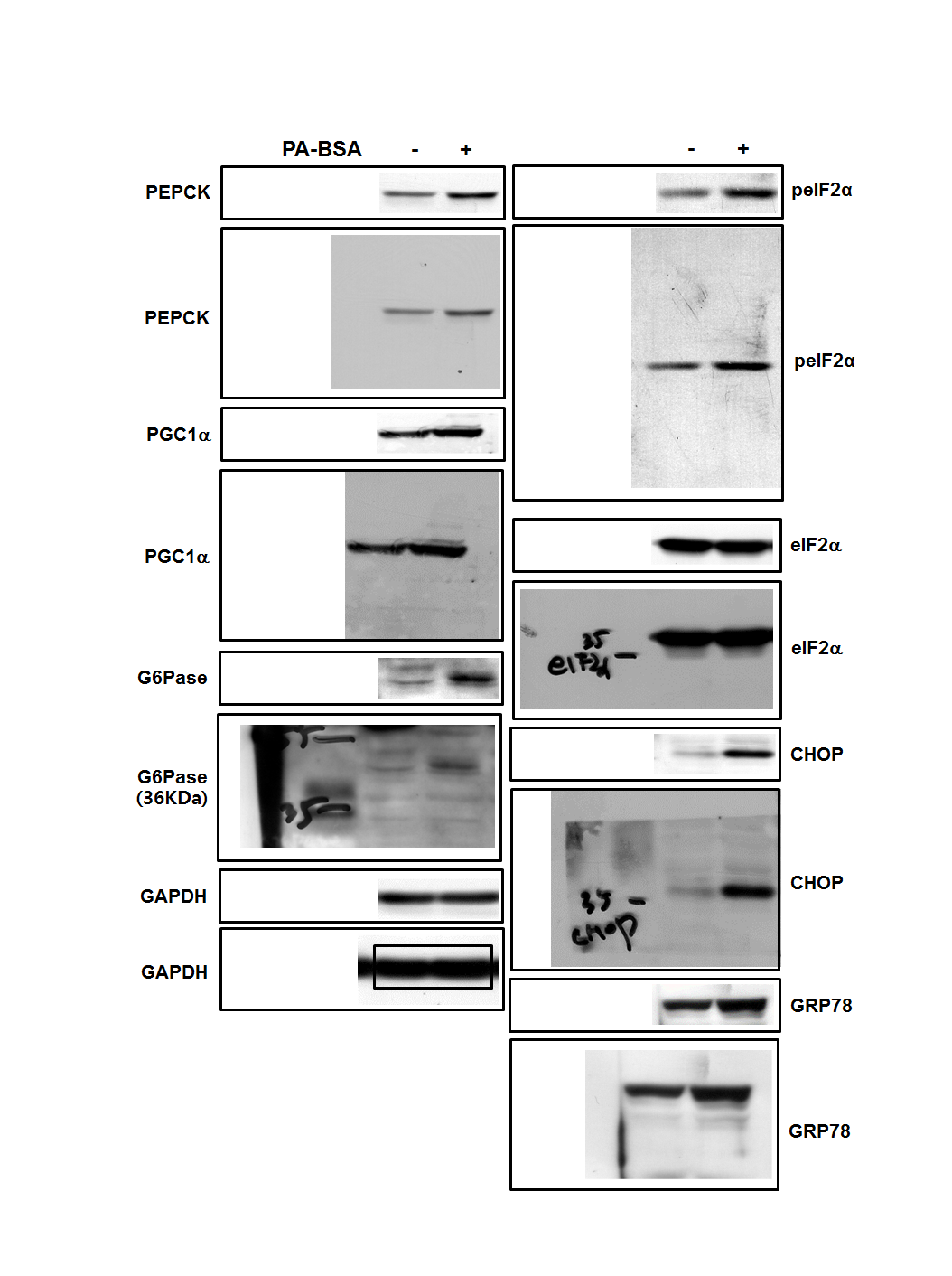
**

Supplementary Figure 2. Middle-length blots and two exposures of Figure 5
